# Climate of Egypt

**Published:** 1861-09

**Authors:** 


CLIMATE OF EGYPT.—Upper Egypt is just now attracting a good deal of attention as a resort for invalids. Dr. Uhle, of the University of Jena, recently published a small *brochure* upon the subject, in which he states that the atmosphere is extremely pure, dry, and exhilarating. The heat of the air never falls at noon below 54·5° of Fahr., and never rises above 86°. The weather is generally fair and cloudless, and there is hardly a day in the winter in which the patient cannot take out-of-door exercise.

